# Effects of indirect plant–plant interaction via root exudate on growth and leaf chemical contents in *Rumex obtusifolius*

**DOI:** 10.1080/15592324.2022.2050628

**Published:** 2022-03-23

**Authors:** Haruna Ohsaki, Akira Yamawo

**Affiliations:** Department of Biological Sciences, Faculty of Agriculture and Life Science, Hirosaki University, Hirosaki, Japan

**Keywords:** Phenotypic plasticity, secondary metabolite, belowground interaction, neighbor recognition

## Abstract

Belowground plant–plant interactions can affect the concentrations of leaf chemicals, but the mechanism is not clear. Here, we investigated the effects of intra- and interspecific root exudates on the growth and leaf chemical content of *Rumex obtusifolius*. Seedlings of *R. obtusifolius* were grown with exposure to root exudates collected from other *R. obtusifolius* plants or from *Trifolium repens, Festuca ovina*, or *Plantago asiatica* plants, and the total phenolic, condensed tannin, dry biomass, and chlorophyll contents of the leaves were examined. The root exudates from conspecific plants had no effect on the total phenolic, condensed tannin, and chlorophyll contents of the leaves but did significantly reduce the dry leaf biomass. Root exudates from heterospecific plants had different effects depending on the species. These results were different from the results of a previous study that examined the effects of direct plant–plant interaction in *R. obtusifolius*. Thus, indirect interaction via root exudates induces different effects in leaves from direct interaction.

## Introduction

Plant–plant interactions are an important part of terrestrial ecosystems because they affect not only the outcome of competition between plants,^1,2^ but also functional leaf traits,^3–7^ hervibory,^8,9^ and herbivore distributions.^9^ In a previous study, we experimented with *Rumex obtusifolius* (Polygonaceae) to examine the effects of intraspecific, interspecific, and no belowground direct interactions on leaf chemical content and herbivore distribution.^9^ Plants exposed to intraspecific direct interaction had increased total phenolic and condensed tannin concentrations in their leaves, and induced a concentrated specialist herbivore distribution on the leaves. A wide variety of plant parts (e.g., leaves, roots, and seeds) and media (e.g., volatile chemicals, nonvolatile chemicals, light, and soil microorganisms) are involved in plant–plant interactions.^10^ Detailed elucidation of the mechanisms of plant–plant interactions would greatly improve our understanding of not only how these interactions affect leaf traits, but also how they affect terrestrial ecosystems.

Kin and self-discrimination in plants occurs via root exudates.^11–13^ Previous study reported that some plant species develop more roots when growing in the vicinity of a non-self plant than when growing in the vicinity of a self plant.^13^ Moreover, similar root behavior was observed in an experiment in which root exudate reduced both root growth and clonal reproduction in non-self, competitor plants. On the basis of these findings, we hypothesized that, if these results depend on indirect interactions mediated by root exudates, *R. obtusifolius* leaf chemical contents, which are linked to herbivore distribution in an ecosystem,^9^ depend on the recognition of conspecific neighbors via root exudates . More specifically, we hypothesized that the concentrations of secondary chemicals in leaves of *R. obtusifolius* are increased in response to exposure to root exudates from plants of the same species, but not to those from plants of other species. Conversely, if our previous results^9^ depended not only on indirect interactions mediated by root exudates but also on other interactions, such as direct contact and resource competition, the effects of these indirect root-exudate-mediated interactions on leaf traits may differ from those of direct plant–plant interactions.

## Materials & methods

### Cultivation

In September 2016, around 300 seeds of *R. obtusifolius* were collected from two plants about 2 km apart in fields in Hirosaki City, Aomori Prefecture, Japan. As interspecific neighbors, we used *Plantago asiatica* L. (Plantaginaceae), *Trifolium repens* L. (Fabaceae), and *Festuca ovina* L. (Poaceae), which are the dominant competitors of *R. obtusifolius* in Japan.^14^ Native to Europe, the perennials *T. repens* and *F. ovina* now grow worldwide. *Plantago asiatica, T. repens*, and *F. ovina* are sympatric with *R. obtusifolius* in Japan. A hundred seeds of *P. asiatica* were collected from two plants in a field in Aomori Prefecture. A hundred seeds of *T. repens* were collected from plants in a field in Saga Prefecture. Commercially available *F. ovina* seeds (Kaneko Seeds Co., Gunma, Japan) were bought. All seeds were stored in a refrigerator at 4°C until use. On 3 September 2017, all seeds from each species of mother plant were mixed and sown on the surface of wet sand (2 cm deep) held in a container. The containers were then kept in a growth chamber at 25°C under a 12-h light/dark cycle until the plants had developed their first true leaves, then healthy of them were randomly used in the subsequent experiment.

To obtain donor plants, we filled 350 plastic pots (10.5 cm diameter × 9 cm high) with sand (Sunday Co., Ltd., Aomori, Japan), and on 13 September 2017 we planted seedlings of each species individually in 280 of these pots (1 seedling/pot; 70 pots/species). The remaining 70 pots were left unplanted as controls. All pots were watered once a day for 30 days. To obtain recipient plants, on 13 October 2017 we planted *R. obtusifolius* seedlings individually in 350 pots containing sand. After sowing, the pots were arranged randomly in growth chambers and maintained at 25°C under a 12-h light/dark cycle. Two days after planting, 0.5 g of solid fertilizer (ammonia nitrogen, 8.0%; soluble phosphorus, 8.0%; water soluble potassium, 8.0%; Nichiryunagase Co., Ltd., Japan) was applied to each pot. The experiment duration, water and soil conditions, and growth conditions were similar to those in the direct interaction experiment in our previous work.^9^

Each of the 350 recipient plants was paired with one of the 350 donor plants and labeled accordingly. Each day at 12:00, 40 mL of distilled water was added to the top of the sand containing the donor plant and 20–25 mL of root exudate was collected from the bottom of the pot, and then added to the top of the sand containing the recipient plant. As smaller plants could not produce enough root exudates for the experiment, we planted the donor plants a month earlier than the recipient plants. All donor plants were perennial species, and *R. obtusifolius* produces seeds every year. Consequently, it is normal for seedlings of *R. obtusifolius* to be surrounded by mature conspecific plants and other plant species in the field, and therefore their roots are exposed to those plants’ exudates.

Thirty days after planting the recipient plants, we obtained a total of 233 recipient plants (control, *N* = 66; intraspecific treatment, *N* = 45; interspecific treatments: *T. repens, N* = 42; *F. ovina, N* = 58; *P. asiatica, N* = 22).

### Measurement of leaf chemical contents

At 30 days, the leaves of the recipient plants were harvested. First, chlorophyll content in the most recently fully expanded leaves was determined. Chlorophyll content reflects the plant’s nitrogen concentration and has been found to indirectly affect vertebrate and invertebrate herbivore survival and distribution .^15,16^ Therefore, we measured this to examine changes in nutrient condition in response to exposure to root exudate. Measurements were conducted with a chlorophyll meter (SPAD-502 Plus; Konica Minolta, Tokyo, Japan), which is a commonly used tool for rapid and nondestructive estimation of leaf chlorophyll content; the resulting SPAD values are positively correlated with chlorophyll content.^17^ Each leaf was measured twice – in the central part of the leaf on each side of the main vein – and the average value per a leaf was determined.

Phenolics and condensed tannins are major secondary metabolites in genus *Rumex*^18^ and have been suggested to stimulate feeding by some leaf beetle species.^19,20^ Therefore, we also determined their contents in leaves. After determined, all recipient plants were harvested and dried at 50°C for 3 days. The dried plants were weighed on an electronic balance to the nearest 0.1 mg, and the total phenolic and condensed tannin contents of the leaves were determined.^21,22^

### Statistical analysis

All statistical analyses were performed in R v. 4.0.2 software.^23^ All data met the statistical assumptions of normality and homoscedasticity according to the Kolmogorov–Smirnov test. The chlorophyll, total phenolic, and condensed tannin contents in leaves were compared among root exudate treatments by using a general linear model with a Gaussian distribution and an identity link followed by an *F*-test; the models included each leaf chemical trait as a response variable and root exudate treatment as the explanatory variable. The false discovery rate correction for multiple comparisons was then applied. All tests were two-tailed, with *P* < .05 considered significant.

## Results and discussion

**Figure 1.**
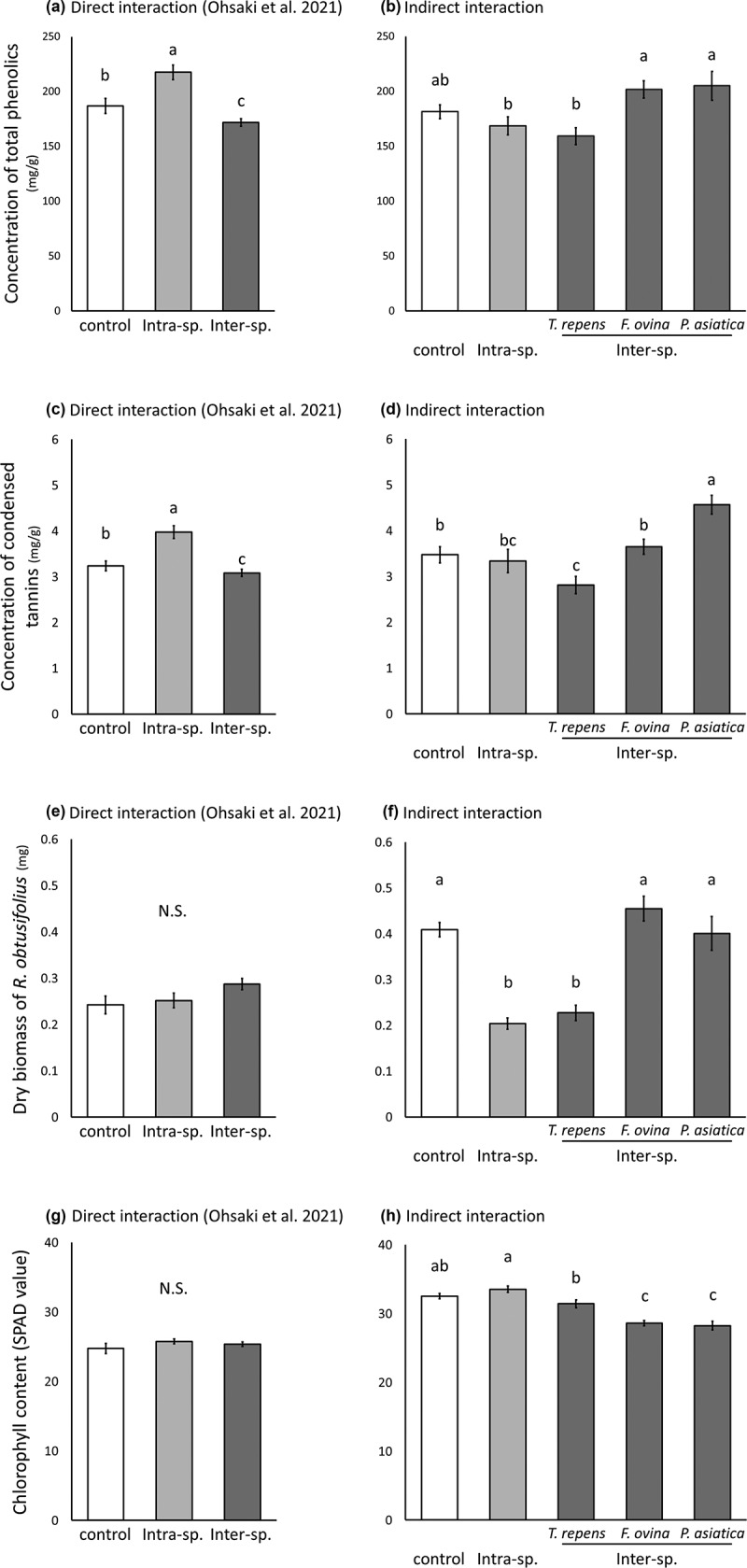
Total phenolic, condensed tannin, dry biomass, and chlorophyll contents in leaves of *Rumex obtusifolius* exposed to (a, c, e, g) direct interaction (grown in same pot)^9^ or to (b, d, f, h) indirect interaction via root exudate (present study). Intra-sp., intraspecific interaction; Inter-sp., interspecific interaction. N.S., non-significant. Bars indicate standard errors. Different letters denote significant differences (general linear model, *P* < .05).

The effect of root exudate on the leaf chemical content of *R. obtusifolius* differed according to the species from which the root exudate was obtained (Figure 1). In *R. obtusifolius* exposed to intraspecies root exudate, the total phenolic and condensed tannin concentrations in the leaves did not differ from those in control leaves (total phenolics: *F* = 1.581, *p* = .211, Figure 1(b); condensed tannins: *F* = 0.217, *p* = .642, Figure 1(d)). This result differs from those of previous study, in which the leaves of *R. obtusifolius* exposed to intraspecific direct interaction had significantly higher total phenolic and condensed tannin concentrations than those in control leaves (Figure 1(a,c)).^9^ Together, these findings indicate that total phenolic and condensed tannin concentrations in the leaves of *R. obtusifolius* are altered in response to direct, but not indirect, intraspecies interaction. In our previous study, *R. obtusifolius* exposed to intraspecific direct interaction had increased total phenolic and condensed tannin concentrations in the leaves, and this induced a concentrated specialist herbivore distribution on the leaves.^9^ If these chemicals directly induce a concentrated distribution of leaf beetles, then indirect interaction with *P. asiatica* may affect this distribution in the field.

The dry leaf biomass of *R. obtusifolius* exposed to intraspecies root exudate was significantly less than control leaves (*F* = 91.413, *p* = .001, Figure 1(f)), indicating that the root exudate of *R. obtusifoliu*s contains substances that inhibit the growth of conspecific plants.^24,25^ Phenolics and condensed tannins have allelopathic effects and are some of the most abundant allelochemicals in higher plants.^26,27^ *Festuca ovina* and *P. asiatica* are strong competitors of *R. obtusifolius*: grassland plant communities often shift from being *R. obtusifolius* dominant to being *F. ovina* dominant;^28^ and, like *R. obtusifolius, P. asiatica* is a perennial herb that develops a leaf rosette. Leaf chemicals in *R. obtusifolius* inhibit the germination of *F. ovina*,^29^ and *R. obtusifolius* may increase them as a competitive response.

Root exudates often include primary metabolites such as sugars and organic acids.^30^ These metabolites may function as fertilizers, but we did not find more increase of biomass in any condition than control condition (Figure 1(f)). We consider that any fertilization effect was absent, or was canceled by the allelopathic effects of secondary metabolites.

The effect of root exudate on chlorophyll content also depended on the species from which the exudate was collected (Figure 1(h)). Plants exposed to root exudate from *F. ovina* or *P. asiatica* had a significantly higher chlorophyll content than plants exposed to that from *T. repens* or conspecific plants (*F. ovina* vs. control, *F* = 51.949, *p* < .001; *F. ovina* vs. *R. obtusifolius, F* = 65.628, *p* < .001; *F. ovina* vs. *T. repens, F* = 17.266, *p* < .001; *P. asiatica* vs. control, *F* = 34.138, *p* < .001; *P. asiatica* vs. *R. obtusifolius, F* = 45.396, *p* < .001; *P. asiatica* vs. *T. repens, F* = 12.241, *p* < .001). The composition of root exudate is likely to be species specific.^31,32^ Here, the root exudate of conspecific plants reduced dry leaf biomass and that of *T. repens* decreased condensed tannin concentration and dry leaf biomass in *R. obtusifolius*. The significant reduction of chlorophyll content in plants exposed to root exudate from *F. ovina* or *P. asiatica* suggests that these root exudates inhibit the uptake of constituents of chlorophyll (e.g., nitrogen and magnesium) or increase the specific leaf area, which decreases aboveground competition.^33^

Recent studies have pointed out that root exudates or root chemicals alter the soil microbial community and feedback for plant growth and resource allocation.^34–36^ The effects of root exudates on *R. obtusifolius* leaf traits may include those caused by changes in soil microbial composition. To understand more about the specific effects of root exudates, detailed analysis of the compositions of root exudates from different species and their effects on soil microbes are needed.

Taking together these and our previous results,^9^ we conclude that the effects of indirect interaction via root exudates are different from those of direct interaction. In short, *R. obtusifolius* may compete more strongly with *F. ovina* and *P. asiatica* by increasing the content of total phenolics and reducing that of chlorophyll in leaves. These results suggest that *R. obtusifolius* seedlings recognize other species via root exudates and express a competitive response, as do other species^37^. If so, leaf traits in *R. obtusifolius* are modulated in space. For example, when plants are close together, the leaf chemical contents are affected by direct interactions, whereas when plants are farther apart, they are affected by indirect interactions. These results highlight the importance of distinguishing between direct and indirect belowground interactions between plants for understanding the effects of plant–plant interactions not only on the plants themselves, but also on the herbivores.
